# *Cryptococcus gattii* Genotype VGIIa Infection in Man, Japan, 2007

**DOI:** 10.3201/eid1607.100106

**Published:** 2010-07

**Authors:** Koh Okamoto, Shuji Hatakeyama, Satoru Itoyama, Yoko Nukui, Yusuke Yoshino, Takatoshi Kitazawa, Hiroshi Yotsuyanagi, Reiko Ikeda, Takashi Sugita, Kazuhiko Koike

**Affiliations:** Author affiliations: University of Tokyo Hospital, Tokyo, Japan (K. Okamoto, S. Hatakeyama, S. Itoyama, Y. Nukui, Y. Yoshino, T. Kitazawa, H. Yotsuyanagi, K. Koike);; Meiji Pharmaceutical University, Tokyo (R. Ikeda, T. Sugita)

**Keywords:** Cryptococcus gattii, central nervous system infection, pulmonary cryptococcosis, genotype, multilocus sequence typing, parasites, Japan, dispatch

## Abstract

We report a patient in Japan infected with *Cryptococcus gattii* genotype VGIIa who had no recent history of travel to disease-endemic areas. This strain was identical to the Vancouver Island outbreak strain R265. Our results suggest that this virulent strain has spread to regions outside North America.

*Cryptococcus neoformans* and *C*. *gattii* are closely related species of yeast; *C*. *gattii* was previously classified as *C. neoformans* var. *gattii* (*1*). Although both species cause pulmonary or central nervous system infections, they differ in their ecology, epidemiology, and pathobiology. *C. neoformans* is the most common *Cryptococcus* spp. worldwide and mainly affects immunocompromised hosts. In contrast, *C*. *gattii* mainly affects immunocompetent hosts and often forms mass-like lesions (cryptococcomas).

Multilocus sequence typing can be used to divide this species into 4 molecular genotypes, VGI–VGIV, which differ in epidemiology and virulence (*1**,**2*). *C*. *gattii* was believed to be restricted to tropical and subtropical areas such as Australia, Southeast Asia, and South America (*1*). However, in 1999, a *C. gattii* infection outbreak occurred on Vancouver Island, British Columbia, Canada (*3*), which has a temperate climate.

During the Vancouver Island outbreak, most human, animal, and environmental isolates obtained belonged to VGIIa (major genotype, 90%–95% of isolates) and VGIIb (minor genotype, 5%–10% of isolates) (*2**,**3*). These strains have now spread to mainland British Columbia and the Pacific Northwest region of the United States (*1**,**4**,**5*). The potential for further spread of this strain, particularly the VGIIa genotype, is a serious concern because it is highly virulent in mammals and can infect immunocompetent persons (*2*).

We report a case of cerebral cryptococcoma caused by *C. gattii* VGIIa (a strain identical to the Vancouver Island outbreak major genotype strain R265) in a patient from Japan who had no recent travel history to known disease-endemic areas.

## The Patient

In 2007, a 44-year-old man (sign painter) from Japan who was not infected with HIV came to the University of Tokyo Hospital with a 2-month history of headache and loss of right-sided vision in both eyes (right homonymous hemianopsia). He had had a diagnosis of hyperglycemia 3 years before admission but had declined to seek medical treatment. His medical history was otherwise unremarkable. He was an ex-smoker who rarely consumed alcohol and was not taking any prescription medications (including corticosteroids or other immunosuppressive drugs). He had traveled to Guam in 1990 and Saipan in 1999 and had no other history of overseas travel. He reported exposure to his dog, and he did not spend time in wilderness areas. The patient often worked near construction sites in urban locations.

When hospitalized, the patient was afebrile and had stable vital signs. Physical examination showed agraphia (inability to write), anarithmia (inability to count numbers), right homonymous hemianopsia, and Romberg sign. Laboratory evaluations showed increased glucose (367 mg/dL) and hemoglobin A1c (10.5%) levels. Results of a complete blood cell count and hepatic and renal function tests were within normal limits. Levels of electrolytes and C-reactive protein were also within normal limits. Results of a test for antibodies to HIV-1/2 were negative.

An enhanced brain magnetic resonance imaging scan showed a 4.4 × 4.1 × 3.3–cm lobulated mass in the left occipital lobe with surrounding edema. The lesion had low signal intensity on T1-weighted images, moderate signal intensity on T2-weighted images, and rim enhancement on T1-weighted images after administration of gadolinium (Figure). A chest computed tomography scan showed a 1.8 × 1.2–cm nodule in the left lower lung (S8 segment).

**Figure:**
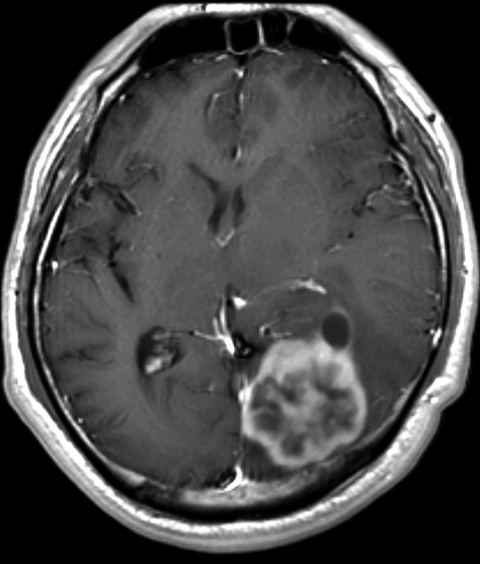
Postcontrast T1-weighted magnetic resononance image of the brain of a 44-year-old man with cerebral cryptococomma in Japan, 2007, showing a rim-enhancing lobulated mass (lower right) with surrounding edema in the left occipital lobe.

The brain mass was completely resected because it was suspected to be a tumor. Pathologic evaluation of the resected specimen showed that the mass was a large abscess containing encapsulated yeast. The yeast could be seen after staining with periodic acid–Schiff and Grocott methenamine silver stains. Specimen cultures were positive for *Cryptococcus* spp. and led to a diagnosis of cerebral cryptococcosis.

The patient was treated with liposomal amphotericin B (4 mg/kg/d for 3 weeks) and flucytosine (100 mg/kg/d for 3 weeks). A cerebrospinal fluid (CSF) sample obtained by lumbar puncture soon after mass resection showed a slightly increased protein level but no leukocytosis or a low glucose concentration. Fungal cultures of the CSF showed negative results. Cryptococcal antigen titers were >512 in serum and 64 in CSF.

After 3 weeks of induction therapy, the patient received consolidation and maintenance therapy (oral fluconazole, 400 mg/d) for 1 year. The pulmonary nodule decreased in size after antifungal treatment, suggesting that this lesion was a pulmonary cryptococcoma. At the 1-year follow-up visit, the cerebral cryptococcoma had not recurred, and the pulmonary nodule had disappeared.

The cryptococcal strain isolated from the brain was identified as serotype B by slide agglutination test (*6*) and identified as *C. gattii* by rRNA gene sequence analysis (*7*). Eleven unlinked loci (*SXI1, IGS, TEF1, GPD1, LAC1, CAP10, PLB1, MPD1, HOG1, BWC1*, and *TOR1*) analyzed by multilocus sequence typing, according to the method of Fraser et al. (*2*), were identical to those of the Vancouver Island major genotype strain R265 (genotype VGIIa) (Table) (*2**,**8**,**9*). MICs of this isolate were 0.125, 0.5, 1.0, and 0.011 µg/mL for amphotericin B, flucytosine, fluconazole, and itraconazole, respectively.

**Table:** Multilocus sequence typing profiles of 2 *Cryptococcus gattii* strains, Japan, 2007*

Strain	Multilocus sequence typing profile†										
	*SXI1α*	*IGS*	*TEF1*	*GPD1*	*LAC1*	*CAP10*	*PLB1*	*MPD1*	*HOG1*	*BWC1*	*TOR1*
JP01	18	4	7	1	4	1	1	5	1	1	1
R265	18	4	7	1	4	1	1	5	1	1	1

*JP01, strain from Japan (patient in this study); R265, Vancouver Island genotype VGIIa strain. †GenBank accession nos. for multilocus sequencing typing alleles: *SXI1α*_18, DQ096308; *IGS_*4, DQ096314; *TEF1_*7, DQ096364; *GPD1_*1, DQ096377; *LAC1_*4, DQ096400; *CAP10_*1, DQ096416; *PLB1_*1, DQ096343; *MPD1_*5, DQ096334; *HOG1_*1, DQ096456; *BWC1_*1, DQ096428; *TOR1_*1, DQ096470.

## Conclusions

Japan has not been considered a *C*. *gattii*–endemic area. There have been only 2 reports of *C. gattii* infections in Japan; both infections apparently originated in Australia (*10**,**11*). Retrospective surveillance studies found no evidence of *C*. *gattii* infection or colonization in Japan (*12*; Ministry of Health, Labor and Welfare of Japan, 2003, unpub. data). Although the source of the infection in the patient reported here has not been identified, it appears to have originated in Japan. Infections with *C*. *gattii* have not been reported in the places the patient had visited (Guam and Saipan) (*13**,**14*). Analysis of persons who traveled to Vancouver Island indicated that the median incubation period of *C. gattii* infection is 6–7 months (range 2–11 months) (*13*), although an incubation period of 13 months has been reported for 1 patient (*14*).

*C*. *gattii* genotype VGIIa may have been present in the Pacific Northwest region of the United States long before the Vancouver Island outbreak (*2**,**4*). However, no human cases were identified in the United States until January 2006 (*4**,**5*). Genotype VGIIa has now spread from Vancouver Island to mainland British Columbia and the Pacific Northwest region of the United States, possibly because of human activity or animal migration (*1*). However, this genotype has not been reported in any other region, although similar isolates have been obtained from South America (*1*) and from patients who had traveled to affected areas (*1**,**8*). Thus, our findings indicate possible global dispersal of this strain.

Our results suggest that *C. gattii* VGIIa genotype may be spreading. In North America, this genotype has been isolated from various environmental specimens such as tree surfaces, soil, water, and air (*1**,**4*). Soil is believed to be a major potential reservoir of this organism (*1**,**15*). Thus, ecologic and environmental studies on *C*. *gattii* in Japan are needed to determine likely reservoirs and to improve understanding of *C*. *gattii* epidemiology. Although many clinical laboratories in Japan currently do not differentiate between *C*. *neoformans* and *C*. *gattii* infections, identification of cryptococcal isolates to the species level, especially in apparently immunocompetent patients, is needed.
